# Attentional biases to faces expressing disgust in children with autism spectrum disorders: an exploratory study

**DOI:** 10.1038/srep19381

**Published:** 2016-01-13

**Authors:** Xin Zhao, Peng Zhang, Li Fu, Joseph H. R. Maes

**Affiliations:** 1 Behavior Rehabilitation Training Research Institution, School of Psychology, Northwest Normal University, 967 East Anning Road, Lanzhou, 730070, China; 2 Donders Institute for Brain, Cognition and Behaviour, Centre for Cognition, Radboud University, PO. Box 9104, Nijmegen, 6500 HE, The Netherlands

## Abstract

Previous studies on attentional bias towards emotional faces in individuals with autism spectrum disorders (ASD) provided mixed results. This might be due to differences in the examined attentional bias components and emotional expressions. This study assessed three bias components, hypervigilance, disengagement, and avoidance, using faces with a disgust, happy, or neutral expression in a dot-probe and external cuing task in 18 children with ASD and 21 typically developing (TD) children. The children with ASD initially displayed hypervigilance towards the disgust faces, followed by a general tendency to avoid looking back at the spatial location at which any face, irrespective of its emotional expression, had been presented. These results highlight the importance of differentiating between attentional bias components in research on ASD.

Autism Spectrum Disorders (ASDs) refer to a group of neurodevelopmental disorders characterized by persistent deficits in social communication and interaction, as well as repetitive behavioural patterns and restricted interests^1^. One of the symptoms that might underlie the social communication and interaction deficits are difficulties in recognizing emotional facial expressions. For example, relative to typically developing (TD) individuals, individuals with relatively strong autistic traits or ASD generally display such difficulties, specifically for one or more of the basic negative emotions (fear, anger, sadness, and disgust^2^,^3^). These difficulties may be based on less automatic and more shallow face processing in individuals with ASD compared to TD individuals^4^,^5^. However, the results are somewhat mixed, with some studies reporting no recognition difficulties^6^. These mixed results may be partly due to methodological differences between studies. One of these concerns stimulus presentation parameters, such as using cartoon-like or real photos of faces, using static or dynamic stimuli, and the intensity of the displayed emotions^7^. Another reason might be linked to the possibility that some individuals with ASD may use compensatory strategies to reach adequate performance in experimental face recognition tasks^6^. Moreover, when looking at emotional faces, infant and adult individuals with ASD have been found to display decreased viewing times for specific parts of the face relative to their TD counterparts^8^, although the results are mixed in this literature as well^9^. Finally, relative to TD individuals, individuals with ASD show deviant patterns of neural responses to emotional faces in general, and fearful faces in particular^10^,^11^,^12^.

In addition to facial emotion recognition deficits, ASD is also characterized by atypical patterns of attentional bias for facial versus non-facial (i.e. non-social) stimuli. Specifically, when given a choice to look at non-social stimuli (objects) or social stimuli (faces, persons), individuals with ASD display a disproportionate attentional bias towards the objects^13^,^14^, or do not show the same attentional bias towards the social stimuli typically seen in TD individuals^15^,^16^. This bias is especially strong when the objects are related to the circumscribed interests of the ASD individual, at least in preschool and school children^17^,^18^.

The attentional bias in the studies referred to above concerned a preference for looking at non-social compared to social stimuli in ASD participants, when these stimuli were simultaneously presented. However, a distinction can be made between three different component processes of attentional bias^19^,^20^,^21^. The first process concerns a rapid and automatic attentional attraction by a stimulus, also termed engagement or hypervigilance. The second concerns the tendency to dwell in the spatial location at which a stimulus is presented, also termed (lack of) disengagement. Finally, attentional bias further may involve a shift or avoidance component, reflecting the shifting of attention away from a stimulus. These three components have their own neural bases^19^. To our knowledge, so far, very little research has been directed at examining these different bias components in the framework of the processing of facial emotions versus non-social stimuli in ASD. One study^16^ used brief stimulus presentation times in order to specifically capture the early engagement process and found an attentional bias for faces relative to non-social images in 16–47 year-old TD but not ASD individuals. Another study^22^ specifically examined the disengagement process in toddlers, who were presented non-social or face stimuli that were followed by a peripheral target stimulus, and found that the TD toddlers remained looking at central face stimuli longer than the toddlers with ASD did (reflecting less disengagement).

The present study was intended to further explore possible attentional biases for faces in children with ASD. However, rather than presenting a choice between looking at facial versus non-facial stimuli, we were specifically interested in potential differences in attentional bias towards faces with different emotional expressions. At least in TD children and adults, attentional biases (stronger engagement and/or less disengagement) have been observed for faces displaying the threat-relevant emotions of anger and fear, relative to happy or neutral faces^23^,^24^, although such biases may be primarily present in individuals with anxiety disorders^25^. The present study assessed whether such attentional biases can also be found in children with ASD. To our knowledge, there are not many studies that directly speak to this issue, and the few published studies reveal mixed results. For example, in one study^26^ it was found that adults with ASD show faster detection of (schematic) angry faces (presented among happy faces) than happy faces (presented among angry faces) in a visual search task (at least under standard presentation task conditions), suggesting hypervigilance to angry faces. However, when faces expressing different emotions were presented in the framework of an emotional Stroop task^27^ it was found that adult participants with ASD display interference effects with respect to all facial stimuli, regardless of emotional expression, whereas TD individuals specifically display stronger interference effects for male faces with angry compared to neutral expressions. In a study using a visual cueing task with angry, sad, happy, and neutral faces, both TD and ASD adults showed an overall attentional bias towards the happy and angry faces but not sad faces^12^. Finally, a recent study using a cuing task with angry, happy, and neutral faces found evidence in both ASD and TD children for an attentional bias towards the angry and happy faces, although the results of targeted analyses directed at the significance of these biases were not reported^28^. Moreover, the study design used in the latter two studies did not allow for differentiating between the different components of attentional bias. A differentiation between hypervigilance and disengagement was possible in a recent study using angry, happy, and neutral faces^29^. For both TD and ASD children, this study did not find evidence of hypervigilance or lack of disengagement with respect to the angry and happy faces. In sum, evidence for attentional biases in ASD, favouring initial attention and/or (a lack of) disengagement with respect to negative and threatening and/or positive faces relative to neutral faces is mixed.

Given the few previous studies and the mixed results, in the present study we examined the three components of attentional bias in children with ASD towards faces expressing the emotion of disgust relative to those with a happy or neutral expression. The reason for choosing disgust as target emotion, which has never been examined in this framework before, was to maximize the chance of finding attentional bias effects in the attentional tasks used (see below). By doing so, the present study could provide further evidence that children with ASD are capable of displaying differential behavioural attentional biases to faces with different emotional expressions. At least some aspects of the emotional expression of disgust are linked to aversive interpersonal contacts and thus, like anger, contain a social aspect^30^,^31^. The ASD children examined in the present study are likely more socially anxious in general than the TD children^32^,^33^ and, at least among socially anxious individuals, disgust expressions are rated as even more negative than angry expressions^34^. Moreover, compared to other emotions, disgust has been found to be the least well recognized facial emotional expression in high-functioning children and adolescents with ASD^7^, implicating a further clear difference with happy and neutral facial expressions.

We used two paradigms to study attentional biases in emotional face processing. The first paradigm was the dot-probe task^35^, which has been used before in research on face processing in ASD, including some of the studies outlined above^12^,^16^,^28^. In this paradigm, pairs of stimuli, a face with a happy and disgust emotional expression, were first presented, one above and one below a fixation point. This was followed by a target stimulus at the spatial location of one of the two faces, to which the participant had to respond. A faster response to the target replacing the face expressing disgust than the happy face indicates an attentional bias to the former face. One limitation of a standard dot-probe task is that it is impossible to separate the different component processes of attentional bias, although the avoidance component may be reflected in *slower* responding to the target replacing the face displaying disgust than the happy face when using relatively long face-presentation times^19^. The second task was an exogenous (spatial) cuing task^36^. This task can differentiate between hypervigilance, disengagement, and avoidance. Participants fixated a central point located between two rectangles. One of the rectangles was replaced by a stimulus, which was a face with either a disgust, happy, or neutral expression. The face was followed by a target cue presented at either the location of the face (‘valid’ trials) or the other location (‘invalid’ trials), and the participant had to respond as quickly as possible to the target. Hypervigilance for faces displaying disgust would be reflected in *faster* responses on *valid* trials cued by a disgust face relative to those cued by a happy or neutral face. Difficulty of disengagement from the face expressing disgust is reflected in *slower* responses on *invalid* trials cued by the disgust face relative to those cued by the happy or neutral face. Finally, avoidance of the faces expressing disgust is reflected in *faster* responding on *invalid* than *valid* trials that are cued by a face showing disgust. Importantly, of the three components of attentional bias, hypervigilance is held to be the fastest and most automatic process, avoidance the most slow and strategic process, and disengagement a mixture of both automatic and strategic processes^19^. Accordingly, the three components require different times to unfold and systematically manipulating stimulus onset asynchronies (SOAs; time between stimulus and target onset), as was also done in our study, is important for establishing a more complete picture of attention allocation across time^21^. Specifically, relatively short SOAs tap into the early hypervigilance process, whereas relatively long SOAs are required to make the more controlled processes of disengagement and (especially) avoidance detectable.

Because of the relative lack of prior studies, and their mixed results, we did not have strong a priori hypotheses about whether any attentional bias would be present in the children with ASD and the TD children for faces expressing disgust relative to those with a happy or neutral emotional expression, and if so, which attentional bias component(s) would be involved. In this sense, the present study was more exploratory than hypothesis testing.

## Results

### Dot-probe task

**Figure 1:**
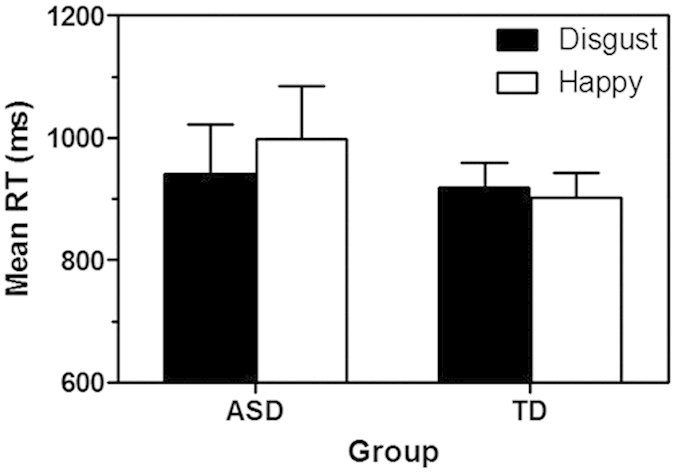
Mean RT (+SEM) on trials during the dot-probe task that were cued by a face expressing disgust or happiness, for ASD and TD children.

The children with ASD reached a significantly lower mean accuracy level (80.8%) than did the TD children (97.8%, *F*(1, 37) = 45.57, *p* = 0.001, *η*_p_^2^ = 0.55). Figure 1 displays the mean (+standard error of the mean, SEM) RT for each group on trials in which the target location was cued by a face with a happy or disgust expression. Neither the main effect of Group (*F*(1, 37) <1) nor Cue Type (*F*(1, 37) = 3.24, *p* = 0.08, *η*_*p*_^2^ = 0.08) was significant. However, the Group × Cue Type interaction was significant (*F*(1, 37) = 9.37, *p* = 0.004, *η*_*p*_^2^ = 0.20), reflecting faster responding in the ASD group when the target had been cued by a disgust than by a happy face (F(1, 17) = 9.46, p = 0.007, η^2^ = 0.36), but no significant RT difference as a function of cue type in the TD group (F(1, 20) <1).

### Exogenous cuing task

Table 1 shows the mean (+standard deviation, SD) RT for each group (ASD, TD), cue type (happy, disgust, neutral), SOA (550, 800, 1100, 1700), and cue validity condition (valid, invalid). The children with ASD reached a significantly lower mean accuracy level (84.1%) than did the TD children (98.3%, *F*(1, 37) = 57.92, *p* < 0.001, *η*_p_^2^ = 0.61). A Group × Cue Type × Cue Validity ANOVA on the RTs for the 550-ms SOA condition revealed significant main effects of Group (*F*(1, 37) = 11.58, *p* = 0.002, *η*_*p*_^2^ = 0.24) and Cue Type (*F*(2, 74) = 4.75, *p* = 0.01, *η*_*p*_^2^ = 0.11), as well as significant interactions between Cue Type and Cue Validity (*F*(2, 74) = 4.25, *p* = 0.02, *η*_*p*_^2^ = 0.10) and between Group, Cue Type, and Cue Validity (*F*(2, 74) = 4.64, *p* = 0.01, *η*_*p*_^2^ = 0.11). The Group × Cue Validity interaction just failed to reach statistical significance (*F*(1, 37) = 4.00, *p* = 0.053, *η*_*p*_^2^ = 0.10). The significant 3-term interaction was examined further with a Group × Cue Type ANOVA for valid (to assess hypervigilance) and invalid (to assess disengagement) trials separately. For *valid* trials, the ANOVA revealed a significant main effect of Group (*F*(1, 37) = 16.52, *p* < 0.001, *η*_*p*_^2^ = 0.31), reflecting slower overall responding in ASD (M = 1131 ms) than TD (M = 858 ms) individuals, and of Cue Type (*F*(2, 74) = 8.70, *p* < 0.001, *η*_*p*_^2^ = 0.19), reflecting overall faster responding to each of the emotional faces than the neutral faces (*p*s < 0.05). The Group × Cue Type interaction term was also significant (*F*(2, 74) = 3.97, *p* = 0.02, *η*_*p*_^2^ = 0.10). Subsequent simple main effect analyses revealed that there was a significant effect of Cue Type in the ASD group (*F*(2, 34) = 8.93, *p* = 0.001, *η*_*p*_^2^ = 0.34) but not the TD group (F(2, 40) < 1). Simple contrast analyses revealed that, relative to the disgust faces (M = 1011 ms), the children with ASD responded significantly slower to the neutral faces (M = 1245 ms; *F*(1, 17) = 25.76, *p* < 0.001, *η*_*p*_^2^ = 0.60), and marginally significantly slower to the happy faces (M = 1138 ms; *F*(1, 17) = 4.25, *p* = 0.055, *η*_*p*_^2^ = 0.20). A Group × Cue Type ANOVA on the data from the *invalidly* cued trials only revealed a main Group effect (*F*(1, 37) = 5.92, *p* = 0.02, *η*_*p*_^2^ = 0.14), reflecting overall slower responding in the ASD (M = 1083 ms) than TD (M = 904 ms) group (other *F*s < 1.47).

The Group × Cue Type × Cue Validity ANOVA for the 800-ms SOA condition only revealed a significant main effect of Group (*F*(1, 37) = 9.76, *p* = 0.003, *η*_*p*_^2^ = 0.21), again reflecting faster overall responding in TD (M = 890 ms) than ASD (M = 1128 ms) participants (other *F*s < 1.71).

The ANOVA using the data from the 1100-ms SOA trials revealed a significant main Group effect (*F*(1, 37) = 13.03, *p* = 0.001, *η*_*p*_^2^ = 0.26) and a significant Group × Cue Type interaction (*F*(2, 74) = 3.86, *p* = 0.03, *η*_*p*_^2^ = 0.10; other *F*s < 3.47, *p*s > 0.07, *η*_*p*_^2^s < 0.05). Subsequent contrast analyses revealed that the interaction reflected significantly faster overall responding to the negative (M = 803 ms) than the neutral (M = 863 ms; *p* = 0.02) and positive (M = 898 ms; *p* = 0.02) faces in the TD but not ASD children (M = 1146, M = 1139, and M = 1105 ms, respectively).

Finally, ANOVA on the data from the 1700-ms condition revealed significant main effects of Group (*F*(1, 37) = 13.06, *p* = 0.001, *η*_*p*_^2^ = 0.26) and Cue Validity (F(1, 37) = 8.12, *p* = 0.007, *η*_*p*_^2^ = 0.18), as well as a significant Group × Cue Validity interaction (*F(*1, 37) = 7.57, *p* = 0.009, *η*_*p*_^2^ = 0.17; other *F*s < 1.35, *p*s > 0.26, *η*_*p*_^2^s < 0.04). The interaction reflected overall faster responding (independent of facial expression) on invalid (M = 1038 ms) than valid (M = 1212 ms) trials for the children with ASD (*F*(1, 17) = 8.15, *p* = 0.01, *η*_*p*_^2^ = 0.32), whereas there was no significant difference in response speed between valid (M = 863 ms) and invalid (M = 860 ms) trials for the TD children (*F* < 1).

## Discussion

The present study assessed whether children with ASD show an attentional bias towards faces displaying the emotion of disgust relative to happy or neutral faces. This emotion has social relevance and has been shown to be relatively difficult to recognize in individuals with ASD. Moreover, we assessed the nature of any attentional bias in terms of the three components of hypervigilance, disengagement, and avoidance. The dot-probe task revealed faster responding in children with ASD to the target when it was presented at the previous location of a face displaying disgust than of a face displaying happiness, reflecting an attentional bias for the faces expressing disgust. However, no such bias was observed in the TD children. The results from the exogenous cuing task suggest that on valid trials within the shortest SOA condition, putatively tapping into a relatively early hypervigilance process, the ASD children displayed an attentional bias towards the disgust faces relative to the neutral and (somewhat less clearly) happy faces. The TD participants again did not display any evidence of attentional bias. However, on invalid trials in this SOA condition, putatively tapping into a disengagement process, there was no evidence of differential disengagement times as a function of facial emotional expression in neither the ASD nor the TD group of children. Analysis of the data of the longest SOA condition, a condition which putatively allows for more controlled attentional processes, such as avoidance, to become visible, revealed general *faster* responding in the children with ASD to the target when *not* presented at the previous location of a face (irrespective of its expression; invalid trials) compared to when presented at the previous location of a face (valid trials). The combined results suggest that, despite the fact that children with ASD displayed an initial hypervigilant response to faces expressing disgust, at later attentional stages they were very well capable of disengaging from such stimuli, and even showed a general tendency to avoid looking at the location at which a face had previously been presented.

The observed initial hypervigilance to the disgust faces, and perhaps also the later avoidance of faces in general, in ASD may be linked to findings of studies on the neuronal correlates of attention and ASD. Specifically, it is suggested that the more bottom-up attentional mechanism implicated in hypervigilance is importantly sub-served by the amygdala. Instead, prefrontal brain structures involved in top-down processes, such as the anterior cingulated and orbitofrontal cortices, are more strongly involved in disengagement and, especially, avoidance. The brain structures and their interconnections involved in these attentional processes in general have also been shown to be specifically involved in face processing^37^. Interestingly, the amygdala is held to be one of the structures primarily implicated in hypervigilance in autism^12^,^38^, displaying typical activation patterns (hyperactivity) and neuroanatomical features (enlargement). Moreover, amygdala enlargement, as well as its hyperreactivity, has been shown to be associated with enhanced (social) anxiety^39^,^40^. Although evidence suggests that anxiety, which is frequently observed in ASD^32^,^33^, may not be associated with attentional biases to faces expressing the threat-related emotion of anger^28^,^29^, it remains to be seen whether this also holds for the emotion of disgust.

The present study failed to find any differential responding to the different types of facial expression in the TD children. Based on prior research suggesting an attentional bias for threat stimuli even in non-anxious individuals^20^,^26^, one could have expected evidence of either hypervigilance, lack of disengagement and/or avoidance of disgust faces relative to happy or neutral faces. However, for a number of reasons, the chance of finding such effects in TD children could have been weakened in the present study. First, the TD children responded significantly faster and more accurate than the ASD children, which is in line with other studies finding slower responding in ASD than TD individuals in spatial orientation tasks in general^41^. The TD children’s higher accuracy rates putatively reflect their better cognitive capabilities in general and executive functioning in particular, and the fast RTs in the TD children might have resulted in floor effects. Second, for the TD children, the disgust faces could have been insufficiently salient or novel as a negative or threatening stimulus, thereby precluding finding any bias effects^25^. Third, the stimulus duration of 500 ms in the dot-probe and cuing tasks might have been suboptimal for detecting early attentional biases, at least in a TD population.

Our choice to adopt a 500-ms stimulus presentation time was based on previous studies that traditionally have used this duration^21^. However, some studies^42^,^43^ suggest that, in the dot-probe task used in a non-anxious population, an early attentional bias (hypervigilance) for threat stimuli is only visible with a shorter presentation time (e.g., in the order of 100 ms). Similar findings of reduced early attentional bias effects for threat stimuli with increasing cue presentation times have been reported with respect to the cuing task^44^. However, the null results with respect to the TD children also suggest that, again at least in a non-anxious population, the 500-ms presentation time was also not long enough for demonstrating the more controlled attentional processes of disengagement and/or avoidance of disgust faces relative to happy or neutral faces. This was even the case in our cuing task in which we also explicitly used different SOA conditions. In our study, these conditions reflected different times of onset of the target, after onset of the cue, with the cue duration itself being fixed. We reasoned that a delayed target onset would allow for later attentional processes (disengagement and/or avoidance) to become visible. This hypothesis was at least partly supported for the ASD children, who in the longest SOA condition, showed significantly faster responding on invalid than valid trials regardless of cue type, reflecting a general tendency to avoid looking back at the previous location of the faces.

The null results with respect to disengagement in the children with ASD seem to be in contrast with previous results suggesting a specific impairment in attentional disengagement in ASD^45^. However, it is important to note that this evidence was based on other types of task, such as tasks in which a target stimulus was simultaneously presented with other stimuli that remained visible and which did not examine attentional processes related to faces. As also indicated in the introduction, in general, the nature of attentional biases (if any) in ASDs strongly depends on the specifics of the task, such as the nature of the contrasting stimuli^41^.

The present study has some limitations. First, the sample sizes were relatively small and it remains to be seen whether the present results are reliable. However, the fact that we found significant attentional bias effects for the ASD group in both paradigms adds to the reliability of these results. Second, the ASD and TD groups differed from one another in overall RTs and accuracy levels and may also have differed in IQ (although we did not have a formal IQ measure). These differences complicate direct between-group comparisons. However, as also indicated in the Method section, including such a measure in the analyses as a covariate would be problematic in this study, given that a compromised IQ may be a feature intrinsic to the disorder^46^. Moreover, given a lack of relevant literature, it is difficult to tell to what extent and in what way differences in general intelligence might affect attentional biases in tasks such as those used in the present study, over and above a positive association between IQ and general response speed. One recent study on the recognition of emotional facial expressions (sad, neutral, and happy) found that male intellectually gifted adolescents showed better face processing, as indexed by RTs and/or event-related potentials (ERPs), than adolescents with an average IQ^47^. However, the ERP differences concerned relatively late processing stages, not the early stages as presumably implied in the hypervigilance effect found in the present study. Moreover, in later processing stages, the gifted adolescents showed a stronger bias (larger ERP amplitudes) towards the negative (sad) faces than the participants with an average IQ. If anything, the latter finding suggests a positive association between IQ and magnitude of attentional bias for negative facial expressions, which would work against obtaining the hypervigilance results found in our study (the autism group, presumably having a lower IQ, showing a stronger bias towards the negative facial expression than the control group, presumably having a higher average IQ). Finally, and perhaps most importantly, the main results of our study concerned within-ASD group differences which are not sensitive to between-group differences. A third limitation is that the ASD children in our study might not be representative of the autism spectrum. Relatedly, due to the requirements of the experiment, all children in the ASD group had relatively intact social and cognitive abilities, implicating an overrepresentation of high-functioning autism. Also, it remains to be seen whether the present results also hold for adolescents and adults with ASD. However, in this respect it is relevant to note that, although the age range of the sample in our study also included adolescence, using the factor age as covariate in our analyses did not change the significance of the critical interaction effects and in the follow-up analyses that were motivated by these interactions, age did not significantly interact with the simple main effect of interest. These results suggest that the present findings are invariant to age. A final limitation is that at present it is unknown what aspect(s) of the disgust faces is (are) responsible for the initial hypervigilance, which could be due to, for example, their relative novelty or difficulty to recognize, or their specific emotional content. Future studies should follow-up on the present preliminary findings, using other ASD sub-populations, other, more direct, tests for attentional biases, like eye-tracking tasks, and including a larger set of different emotional face expressions.

Despite these limitations, the present results expand our knowledge on face processing in ASDs. They suggest that, in studies on this topic, it is of critical importance to distinguish between different components of attentional bias. Our results suggest that, at least in single- and two-face conditions, children with ASD display an early attentional bias towards faces expressing the emotion of disgust. This hypervigilance seems to be followed by a normal general disengagement ability. However, we found evidence that following disengagement, at least when given enough time to allow the corresponding process to become visible, the ASD children have a general tendency to remain ‘disengaged’, in the sense of further avoiding looking back at the location at which any face stimulus had been presented, regardless of its emotional content. Especially the latter phenomenon may preclude the possibility to habituate to faces in general^48^, and to further process such faces, thereby maintaining relative difficulties in their recognition and interpretation.

## Method

### Participants

#### ASD group

Twenty five children with ASD were recruited from three special education schools. All children were from the Han population. Informed consent forms were signed by their parents. The study was approved by the Institutional Review Board from the School of Psychology at Northwest Normal University. All experimental manipulations were performed in accordance with the approved guidelines. Each child was diagnosed with ASD based on a clinical assessment incorporating the Childhood Autism Rating Scale (CARS^49^; see below). Children with a genetic disease, major sensory or motor nerve damage, or disability were excluded. All children had normal or corrected-to-normal vision. Further inclusion criteria were: (a) at least average intelligence and being able to understand the task instructions; (b) capability of recognizing emotional faces (both real and cartoon pictures); (c) being able to have a simple conversation with an acquaintance and understanding the meaning of simple physical gestures; (d) being able to concentrate on an ongoing task for more than 15 min. Assessment of these criteria was not based on formal tests, which would be rather lengthy and possibly could have evoked distress and an unwillingness in at least some of the children to cooperate with the main tasks. Therefore, we decided to have the various inclusion criteria assessed by way of a more-or-less structured interview of the children by a teacher in the rehabilitation centre with whom the children were familiar. One drawback of not having a formal measure of intelligence (IQ) is that one cannot statistically assess the extent to which any between-group differences, if found, are driven by an IQ difference between groups rather than by autism per se. However, even if we would have had a reliable IQ measure, including it in the analyses as a covariate is problematic in cognitive studies of neurodevelopmental disorders^46^. Specifically, if the covariate, in this case IQ, is an attribute of the disorder, or at least of some important features of the disorder, as is likely the case in autism e.g.,^50^, it is meaningless to try to ‘control’ for it. A priori exclusion criteria based on task performance were: (a) not passing the practice phase of one of the two tests; (b) having an accuracy level of less than 65%; (3) having an exceptionally short (<300 ms) or long (>3000 ms) response time on more than 50% of the trials. The data of seven children were not included based on these exclusion criteria. The mean age of the remaining 18 children (13 boys) was 12.5 years (SD = 2.1, range = 10−16). The mean CARS score of the children was 32.8 (SD = 2.5, range = 30−38), implicating mild to moderate autism (see also below).

#### TD group

Twenty-two children from the Han population were recruited from grades 4−8 from an elementary school. A signed informed consent was provided by their parents. All children had normal or corrected-to-normal vision and none had a neurological disease. The data of one child was excluded due to an accuracy level of less than 65%. The mean age of the remaining 21 children (11 boys) was 12.2 years (SD = 1.1, range = 10−14). The age of the children in the TD group did not significantly differ from that in the ASD group (F < 1). The children were also rated according on the CARS and each child had the minimum score of 15, implicating the absence of any autism-related characteristics.

### Measures and tasks

#### Childhood Autism Rating Scale

We used a Chinese version of the CARS. The CARS consists of 15 scales, each reflecting a specific feature of autism. An adult rates each child for the presence of the features using a 4-point scale (1 = normal for the child’s age; 4 = severely abnormal). Midpoint scores of 1.5, 2.5, and 3.5 may also be used. This implicates a minimum and maximum score of, respectively, 15 and 60. A score of 30−36 reflects mild to moderate autism; a score of >36, in combination with a rating of ≥3 on ≥5 subscales, implicates severe autism. Assessment of the CARS was conducted by assistants in the children’s rehabilitation institutions.

#### Dot-probe task

We employed 14 grey-scale face images (half of which were female) with a happy, neutral, or disgust emotional expression from the Chinese Facial Affective Picture System^51^. All faces used in this, and the following, task were well-validated: a high percentage of reviewers had rated each of the faces as displaying the corresponding emotion. Moreover, the faces with the different emotions did not significantly differ on an intensity dimension using a scale from 1 (lowest) − 9 (highest; data available from corresponding author upon request). All pictures had been modified by Photoshop 7.01 into the size of 10.8 × 12.7 cm with the same luminance. The dot-probe task was presented using E-prime 2.0. During each trial in the practice phase of the task, a white central fixation cross appeared for 500 ms, followed by an empty black screen for a random time between 400−800 ms. Next, two identical pictures (each 4 × 5 cm) of one person with a neutral facial expression were shown for 500 ms. The pictures were displayed one above the other in the vertical central midline, with 4 cm in between the pictures. After the pictures disappeared, a blank screen was shown for a random time between 400−800 ms. Finally, a target stimulus (4 × 5 cm) was presenting randomly at one of the locations of face pictures. Participants were asked to press either the left or right mouse button, depending on the type of target. The target picture consisted of a coloured drawing of either ‘Pleasant Goat’ or ‘Grey Wolf’, two characters from a popular Chinese animated television series. Specifically, they had to press the left and right button in case of, respectively, Pleasant Goat and Grey Wolf. In case the response was correct, the participant received positive feedback in the form of a picture of a yellow smiley with the words “Good job” written underneath. If the response was incorrect, a crying smiley was shown with the words “You will do better next time”. The feedback screen was shown for 500 ms and the next trial started immediately thereafter. The practice phase consisted of 16 trials, 2 (paired) presentations of each of 8 persons. The trials in the experimental phase of the task were identical to those of the practice phase, except that two pictures were shown of the same person, one picture displaying a happy emotion and the other one disgust. The experimental phase consisted of 96 trials. Specifically, in each block of 24 trials, each of the 6 persons (for each person: one with a happy and one with a disgust emotional face) was presented 4 times, for a total of 4 trial blocks. The location of the disgust face (top or bottom of the screen) and the location and nature of the target stimulus were counterbalanced across trials. There was a break between the experimental trial blocks and the child could end a break by pressing the Q key on the keyboard. The task lasted about 15 min.

#### Exogenous cueing task

We used an adapted version of Posner’s (1980)^36^ paradigm. Four faces with a neutral expression were used during the practice phase of the task. Each trial during this phase commenced with a 500-ms central fixation cross, followed by two 4 × 5-cm grey squares presented to the left and right of the fixation cross, 7 cm apart.

A neutral face was then presented for 500 ms at the same spatial location as one of the two grey squares. After an interval of 50, 300, 600, or 1200 ms, implicating a stimulus onset asynchrony, SOA, of respectively 550, 800, 1100, or 1700 ms, a target stimulus was presented at either the location of the previous grey square or the neutral face. The target stimulus, the matching of target to response, and the feedback stimuli were as described for the dot-probe task. Each of the four neutral faces was presented three times, implicating12 practice trials. The location of the face cue and the location and nature of the target were quasi-randomly determined during these trials. Each trial during the experimental phase of the task was identical to that during practice except that four faces were used as cue that each could have one of three different expressions: neutral, happy, or disgust. The faces used in this phase had not been used before in the dot-probe task or in the practice phase of the current task. During each block of 24 trials, each of the emotions of each face was presented two times. The location of the face cue and the location and nature of the target stimulus were counterbalanced across trials. Each child received four 24-trial blocks.

### Procedure

All children were first evaluated using the CARS. The dot-probe task was conducted within two weeks after the CARS evaluation. The children were tested individually in a quiet 15 m^2^ lab room. The child was seated in a comfortable chair at a distance of 40 cm from the screen, with a viewing angle of 2°. After reading the instructions, the participant pressed the Q key to start the practice phase of the task. If response accuracy was less than 80%, the practice trial block was repeated maximally three times. A break was introduced in between the 24-trial blocks of the experimental phase, which the child could terminate by pressing the Q key. The total session lasted about 15 min. The exogenous cuing task was performed one week after the dot-probe task following the same procedure as described for the dot-probe task.

### Data analysis

The dependent measure for each task was the response time (RT) to the target stimulus on correct trials. The RT on incorrect trials and RTs shorter than 300 ms and longer than 3000 ms were not considered. The RTs from the dot-probe task were analysed using a repeated measures analysis of variance (ANOVA) with Group (ASD vs. TD) as between-subject factor and Cue Type (location at which the target appeared: happy vs. disgust face) as within-subject factor. The RTs from the exogenous cue task were first analysed with a repeated measures ANOVA, with Group (ASD vs. TD) as between-subject factor, and Cue Type (happy, disgust, or neutral), Cue Validity (valid vs. invalid), and SOA (cue-target SOA: 550, 800, 1100, or 1700 ms) as within-subject factors. Although this analysis did not reveal a significant 4-way interaction effect (*F*(6, 222) = 1.77, *p* = 0.11, *η*_*p*_^2^ = 0.05), based on a univariate test of within-subject effects, we decided to perform a Group × Cue Type × Cue Validity ANOVA for each SOA separately for two reasons. First, the validity of the univariate approach (which we used to evaluate the other within-subject effects) of the test for significance of this 4-term interaction was compromised by a violation of the sphericity assumption, and a more valid multivariate approach revealed a near-significant effect (*F*(6, 32) = 2.25, *p* = 0.061, *η*_*p*_^2^ = 0.30). Second, and more importantly, as outlined in the introduction, the different SOAs are theoretically linked to the expression of different components of attentional bias, with the shorter SOA being associated more with hypervigilance, and the longer SOAs with disengagement and, particularly, avoidance. All statistical tests used *p* < 0.05 as criterion for significance.

## Additional Information

**How to cite this article**: Zhao, X. *et al.* Attentional biases to faces expressing disgust in children with autism spectrum disorders: an exploratory study. *Sci. Rep.* **6**, 19381; doi: 10.1038/srep19381 (2016).

## Figures and Tables

**Table 1:** RT performance on the exogenous cuing task.

	SOA (ms)	Valid						Invalid					
		Happy		Disgust		Neutral		Happy		Disgust		Neutral	
		*M*	*SD*	*M*	*SD*	*M*	*SD*	*M*	*SD*	*M*	*SD*	*M*	*SD*
ASD	550	1138	338	1011	248	1245	291	1102	298	1104	308	1043	304
	800	1189	365	1175	335	1093	329	1106	288	1075	300	1131	281
	1100	1157	365	1193	421	1169	367	1054	345	1099	312	1109	422
	1700	1192	326	1223	357	1223	394	1055	296	994	295	1064	300
NC	550	846	183	840	174	887	199	923	242	865	166	923	238
	800	936	343	846	197	892	236	912	234	877	215	876	231
	1100	874	204	810	190	839	142	922	222	796	186	887	232
	1700	850	172	888	232	850	220	852	216	846	218	881	249
